# New Aspects of Lung Transplantation: A Narrative Overview Covering Important Aspects of Perioperative Management

**DOI:** 10.3390/life13010092

**Published:** 2022-12-28

**Authors:** Julien Fessler, Michaël Finet, Marc Fischler, Morgan Le Guen

**Affiliations:** 1 Department of Anesthesiology and Pain Management, Hospital Foch, 92150 Suresnes, France; 2 University Versailles-Saint-Quentin-en-Yvelines, 78000 Versailles, France

**Keywords:** lung transplantation, COVID-19, cystic fibrosis, hemostasis, extracorporeal membrane oxygenation, pulmonary graft dysfunction, postoperative pain, anesthesiology

## Abstract

The management of lung transplant patients has continued to evolve in recent years. The year 2021 was marked by the publication of the International Consensus Recommendations for Anesthetic and Intensive Care Management of Lung Transplantation. There have been major changes in lung transplant programs over the last few years. This review will summarize the knowledge in anesthesia management of lung transplantation with the most recent data. It will highlight the following aspects which concern anesthesiologists more specifically: (1) impact of COVID-19, (2) future of transplantation for cystic fibrosis patients, (3) hemostasis management, (4) extracorporeal membrane oxygenation management, (5) early prediction of primary graft dysfunction, and (6) pain management.

## 1. Introduction

Anesthesiologists are primarily concerned with intraoperative management, but also with the selection of patients for lung transplantation and with early postoperative follow-up. Such concerns have been recently highlighted in the International Consensus Recommendations for Anesthetic and Intensive Care Management of Lung Transplantation, jointly sponsored by the European Association of Cardiothoracic Anesthesiology and Intensive Care (EACTAIC), the Society of Cardiovascular Anesthesiologists (SCA), the International Society of Heart and Lung Transplantation (ISHLT), the European Society of Organ Transplantation (ESOT), the European society of thoracic surgeons (ESTS), and the American society of transplantation (AST) [1]. Since there are continuous changes in lung transplantation, we have chosen six themes that have evolved recently. Two themes concern patient selection: impact of COVID-19, which has changed surgical activity and created new indications for lung transplantation, and the future of lung transplantation for cystic fibrosis patients with the introduction of new treatments that have significantly improved the respiratory status of many patients who are no longer candidates for transplantation. Two other themes concern intraoperative management: extracorporeal membrane oxygenation management, whose use has become commonplace, but which remains a highly debated subject, and hemostasis management, which is far from uniform among surgical centers. Finally, two themes concern the early postoperative course: pain management, with techniques in full evolution, and early prediction of primary graft dysfunction with the introduction of innovative biological techniques. We have tried to summarize for each of these themes the current problems, the recent data, and the foreseeable evolution.

## 2. Materials and Methods

This literature review was based on the PubMed database using the following keywords: lung transplantation, COVID-19, cystic fibrosis, hemostasis, extracorporeal membrane oxygenation, pulmonary graft dysfunction, and postoperative pain. Original articles, review articles and case reports from 2001 to 2022 were included in the analysis. However, some older but important articles are cited.

## 3. Results

### 3.1. Impact of COVID-19

Many aspects of lung transplantation have been profoundly altered by the COVID-19 epidemic and the ISHLT published recommendations in 2021 (https://ishlt.org/ishlt/media/documents/SARS-CoV-2_Guidance-for-Cardiothoracic-Transplant-and-VAD-center.pdf, accessed on 11 November 2022).

#### 3.1.1. Consequences on Transplantation Activity

The main consequence was a decrease in the number of transplants and greater difficulty in organizing surgeries because of the constraints on the pulmonology, anesthesia, and intensive care teams. The medical teams tried, when possible, to postpone the time of the lung transplant to manage the restrictions of access to the operating room and intensive care unit, on the one hand, and on the other hand to avoid ending up with a recipient at risk of postoperative contamination. Coiffard et al. reported the results of a survey addressed by e-mail to physicians from 180 centers in 35 countries. The main results from 78 centers (15 countries) were reduction in activity (81% of the responders), increased selection of the recipients to only urgent cases (47%) and increased deaths on the waiting list (20%) [2].

#### 3.1.2. Indications for Lung Transplantation

Severe pulmonary forms of complications from COVID-19 infection correspond to two distinct phenotypes: a relatively early-onset acute respiratory distress syndrome (CARDS for COVID-19 acute respiratory distress syndrome) and a later-onset pulmonary fibrosis (PCPF for post-COVID pulmonary fibrosis) [3,4]. 

Lung transplantation has been proposed as a treatment for CARDS as reported by several small series. Eleven patients were transplanted in Korea, 10 of whom were alive after a median follow-up of 322 days [5]. Five patients, including two pregnant women, were transplanted in Detroit (USA), four of whom were alive after a median follow-up of 273 days [6]. Of particular interest is the report of the Austrian experience: 39,485 patients were hospitalized for COVID-19 in Austria between 1 January 2020, and 30 May 2021, of whom 2323 required mechanical ventilation and 183 received extra-corporeal membrane oxygenation (ECMO) support. Among the 106 patients with CARDS referred for lung transplantation, 19 (18%) underwent lung transplantation. All patients were alive at 30-days post-transplant, and 14 (74%) were alive after a median follow-up of 134 days [7]. However, as Cypel and Keshavjee have pointed out, publication bias is possible, where only series with good outcomes are reported [8]. 

Lung transplantation may be a promising treatment for end-stage respiratory failure following PCPF [9,10]. Since fibrotic disease affects around one-third of patients with a severe pulmonary form of COVID-19; this indication could represent a considerable number of patients in the near future [11,12,13,14]. Risk factors for PCPF include notably advanced age, greater severity of illness, longer stay in the intensive care unit with mechanical ventilation requirement, and a history of smoking or alcoholism [11,12,14,15]. Based on the available data, it is likely that most patients with COVID-19 fibrosis will improve or remain stable, but the length of recovery is unclear. Therefore, it appears important to follow up patients with pulmonary fibrosis related to COVID-19 to better understand the evolution of lung damage and to identify potentially modifiable risk factors of evolution into fibrosis disease [16]. 

#### 3.1.3. Conclusions

The COVID-19 pandemic appears perhaps to be on its way out, which should limit the indications for CARDS transplantation. The next few years will show whether indications for PCPF will increase.

### 3.2. The Future of Lung Transplantation for Cystic Fibrosis Patients

The management of patients with cystic fibrosis (CF) has changed considerably in recent years.

CF used to be a major indication for lung transplantation. The Phe508del mutation of the cystic fibrosis transmembrane conductance regulator (CFTR) is present in approximately 80% of patients, 40–50% being homozygous. Molecules targeting the defective CFTR protein, called CFTR modulators, have been developed in recent years: Ivacaftor, then the double combination therapies (lumacaftor-ivacaftor and tezacaftor-ivacaftor) and finally the triple combination therapy (elexacaftor-tezacaftor-ivacaftor) [17,18,19]. Of great interest is the fact that this therapy is responsible for a drastic change in patient follow up: 2.20 (95% CI: −3.26, −1.14) fewer overall healthcare visit-days, 0.16 (95% CI: −0.22, −0.11) fewer inpatient admissions, 0.33 (95% CI: −0.59, −0.07) fewer infection-related visit-days, and 0.78 (95% CI: −1.03, −0.54) fewer antibiotic prescriptions over a 15 week period [19]. A French study recently showed the evolution of patient status toward lung transplantation twelve months after the initiation of elexacaftor-tezacaftor-ivacaftor among patients enrolled in an early access program [20]. This study included 331 patients, median age of 32 (25; 39) years with advanced CF, 65 of them identified as lung transplant candidates at the time of treatment initiation. Two patients were transplanted, one 5 days after initiation of the therapy and the other 2 days later. Clinical improvement occurred rapidly for other patients, leading to lung transplantation no longer being indicated and no death occurred within this cohort of lung transplant candidates, with mild and transient adverse effects. Consequently, it can be recommended that all CF patients with advanced pulmonary disease and a Phe508del mutation be treated with elexacaftor-tezacaftor-ivacaftor before considering listing for transplantation. As a result, the selection of patients for lung transplantation will be significantly changed.

### 3.3. Hemostasis Management

The management of perioperative hemostasis remains an important issue in lung transplantation particularly in some cases when intraoperative hemorrhage is increased (sarcoidosis, retransplantation and patients with prior chest surgeries with dense adhesions). While blood product transfusion has been shown to be associated with poorer outcomes, targeted transfusion, tailored to the patient’s needs and guided by point-of-care coagulation tests, may be of interest.

#### 3.3.1. Transfusion-Related Risk

Perioperative transfusion of allogeneic blood products in both donor [21,22,23] and recipient has been widely associated with an increased risk of primary graft dysfunction (PGD) and 1-year mortality [24,25,26]. While these studies have highlighted an association between transfusion and poorer outcomes, it is difficult to determine whether the impact of transfusion is related to the nature of the products administered or to the bleeding that led to the transfusion. Moreover, we should mitigate the relationship between transfusion and PGD because of the great difficulty in distinguishing between PGD and transfusion-related lung injury (TRALI). Interestingly, Pena et al. have outlined that developing de novo HLA antibodies is known to negatively affect lung transplant outcomes, particularly increasing the risk of chronic lung allograft dysfunction [27] and the Duke anesthesiology team has presented recently the results of an retrospective, observational study which showed that increased perioperative transfusion requirements were associated with the development of new anti-HLA antibodies including Donor-Specific Antibody [28]. Transfusion must be kept to a minimum, but the threshold of hemoglobin is not clearly defined in lung transplanted patients.

#### 3.3.2. Point-of-Care Coagulation Tests Strategy

Goal-directed transfusion strategy based on a point-of-care coagulation test (POCCT) has shown to be of benefit in cardiac surgery [29] and other organ transplantation such as the liver [30,31]. There are, however, few studies evaluating this strategy in lung transplantation. It was first investigated in a retrospective cohort study by Smith et al. They compared two periods on 93 bilateral lung transplantations. The first group received standard of care, and the second received goal-directed transfusion after implementation of a specific algorithm based on ROTEM and platelet aggregometry assays as well as hemoglobin, temperature, pH, and calcium determinations. All lung transplantations were conducted under CPB, and patients under ECMO as a bridge to transplantation or postoperatively were excluded. The POCCT-based strategy showed a significant decrease in blood transfusion with no detectable changes in outcome aside from a small decrease in early postoperative bleeding. They also performed a cost-analysis which demonstrated an overall reduction in cost of transfusion after the introduction of this strategy [32]. The second study was a randomized controlled trial conducted by Durila et al. They compared a transfusion algorithm based on ROTEM to standard care. The study had to be stopped prematurely in the aftermath of an interim statistical analysis, after including only 67 patients, because the results were significantly in favor of the POCCT-based strategy There was a significant decrease in perioperative blood loss, consumption of RBC, and consumption of fresh frozen plasma without deteriorating clot formation in secondary and primary hemostasis. However, this study had a major bias because patients in the POCCT-based strategy group received a fluid support exclusively with 5% albumin solution, whereas fluid support in the other group was based on crystalloids and colloids [33].

#### 3.3.3. Conclusions

A POCCT-based strategy may help tailor transfusion to the patient’s needs and adapt it throughout the surgery. However, in case of an ongoing massive hemorrhagic shock, this tailored strategy may be replaced by a transfusion ratio of 1 RBC pack to 1 FFP pack since Seay et al. observed a pejorative association between a high FFP:RBC ratio and PGD at 72 h postoperatively [34].

### 3.4. Extracorporeal Membrane Oxygenation Management

Although this is one of the major elements of care, management of ECMO is still not consensual and numerous relevant questions remain.

#### 3.4.1. Bridge to Transplantation

Ethical aspects for the indications of ECMO as a bridge to lung transplantation should be clearly identified and discussed in a multidisciplinary meeting, especially in compassionate or borderline situations (patients too frail for transplantation). The technique used in this indication is most often a veno-venous ECMO (VV-ECMO) with a single catheter which can simultaneously drain venous blood from the central circulation (superior and inferior venae cavae) to deliver oxygenated blood directly into the heart [35]. Preoperative ECMO should be considered in case of right heart failure despite optimal medical management, refractory hypoxemia, or hypercapnia. Preoperative ECMO represents a valuable support as an alternative to invasive mechanical ventilation [36] because awake ECMO strategy allows for early ambulation, reduces ventilator-associated pneumonia and prevents skeletal muscle deconditioning [37]. It also provides favorable long-term outcomes [38]. Habertheuer et al. established a quantitative risk score called the “STABLE score”, which helps patient selection as it assesses the risk of post-transplantation in-hospital mortality for patients on ECMO as bridge to transplantation. It includes six factors: recipient’s age, number of days on waiting list, dialysis, transplant center volume, mechanical ventilation, and total bilirubin) [39]. Interestingly, Furfaro et al. drew attention to the fact that among patients on ECMO as bridge to transplantation, those who had primary pulmonary hypertension had a lower transplantation rate than those with obstructive lung disease, cystic fibrosis, and interstitial lung disease [40].

#### 3.4.2. During Surgery

- CPB or ECMO

Two high quality meta-analyses compared, during lung transplantation, CPB, which requires full heparinization, to ECMO, which requires a low heparin dose [41,42]. They found that ECMO was associated with fewer postoperative complications and improved short-term outcomes (lower blood product transfusion requirements, shorter ventilator support, and shorter length of hospital stay). Furthermore, Yeo et al. have reported that ECMO using low dose heparinization does not markedly increase the rate of thromboembolic complications [43]. Of note, there are no large multicenter randomized controlled trials comparing both supports [44]. However, CPB is mandatory in some cases, such as when the patient requires concomitant intracardiac repair, when there is a past surgical history of pneumonectomy, or when it is difficult to clamp the pulmonary artery or left atrium with enough cuff. CPB circuit is an open system, has a venous reservoir with cardiotomy suction lines and consequently a blood–air interface which increases inflammation. Martin et al. introduced the use of a hybrid ECMO-CPB circuit, which can facilitate easy and immediate conversion to full CPB with no interruption of mechanical circulatory support [45].

- Indications for ECMO

The indication for ECMO and the timing of implantation are major topics of interest. Very roughly, there are two opposing schools of thought.

On the one hand, a common approach is to use ECMO only when required, to reduce the incidence of ECMO-related complications. Many high-volume centers have associated ECMO with poorer outcomes and a higher complication rate [46,47,48,49,50]. Intraoperative ECMO can be preoperatively planned or unplanned to face a hemodynamic or a respiratory failure despite optimal medical management. Salman et al. aimed to identify risk factors and cutoff values for known preoperative hemodynamic indicators that can predict the need for intraoperative ECMO support in case of pulmonary fibrosis [51]. The predictive factors retained were hypercapnia, pulmonary hypertension, decreasing cardiac index, and high pulmonary vascular resistance (especially after general anesthesia induction or clamping of the pulmonary artery), a lower body surface area because of the small chest cavity (that increases difficulty in maintaining a good surgical field), and a small graft. Our team investigated risk factors for an unplanned intraoperative ECMO. In addition to the previous cited factors, we also found pulmonary fibrosis [52], and total lung capacity mismatch (downsizing) [48]. Scaravilli et al. revealed that in an exclusive population of cystic fibrosis patients the risk factors were low right ventricle ejection, higher oxygen, low body surface area, and cystic fibrosis related diabetes [50].

On the other hand, the Vienna Lung Transplant Group thoroughly evaluated ECMO in the field of lung transplantation. In a retrospective monocentric cohort study, Hoetzenecker et al. described routine ECMO before first graft revascularization which reduces reperfusion blood flow in the newly implanted graft. They reported a surprisingly low incidence of grade 3 PGD at 72 h (1.3%), but with one-year survival similar to other high-volume centers (86%). The physio-pathological explanation could be that this ECMO strategy was protective to the vulnerable newly implanted graft during the reperfusion phase [53]. However, they also highlighted that postoperative ECMO increased bleeding complications [54].

- VV-ECMO or VA-ECMO

Some teams use VV-ECMO intraoperatively, especially when it was used before as a bridge, and this may be sufficient if there is no hemodynamic problem. In the latter case, it is necessary to switch to a VA-ECMO. 

In the case of intraoperative ECMO assistance, the question of central or peripheral, i.e., femoral, implantation arises. In the case of peripheral VA ECMO, blood perfusing the brain, heart, and upper extremities comes from either the native lungs or from the ECMO flow. These flows can be competitive, leading then to inadequate oxygenation depending on the cardiac output and ECMO flow (Harlequin syndrome also known as North-South Syndrome). In addition, peripheral ECMO decreases the right ventricle preload and increases the left ventricle afterload. Finally, it is also a source of complications in the groin. In the “central configuration”, arterial injection of the ECMO flow is performed through a cannula located at the root of the ascending aorta, so that the ECMO flow is not competitive with the cardiac flow. Thus, central ECMO decreases right ventricular preload and left ventricular afterload. However, the main weakness of this setup is the need to convert it to a “peripheral configuration” if ECMO weaning fails at the end of the procedure. 

After a review of 77 lung transplantations performed in their center from 2009 to 2020, Ruszel et al. advocated the use of central ECMO since they observed a higher survival rate with the use of central ECMO than with peripheral ECMO or without external support. However, they reported an unusually high mortality making their result non-generalizable [55]. As each technique offers its own advantages and given the state of the literature on the subject to date, the choice of ECMO implantation site must be determined by the surgical approach and the expertise of the centers.

#### 3.4.3. Weaning at the End of Surgery

As for intraoperative ECMO implantation, there are two attitudes regarding the maintenance of the ECMO postoperatively: routine maintenance or withdrawal in case of a favorable weaning trial. Interestingly, the Vienna Lung Transplant Group has changed its practice along the past years from advocating routine postoperative ECMO maintenance to ECMO continuation in selected patients at the end of surgery (8% of all cases) [53,56]. Some factors predicting weaning at the end of surgery have been reported: younger donor age, donor high PaO2, and shorter operation time [57]. Obviously, ECMO remains mandatory in case of severe PGD at the end of surgery with increased initial mortality but comparable long-term survival with that of other patients [58].

#### 3.4.4. Conclusions

It seems probably more accurate to adapt the ECMO strategy to personalized factors. ECMO should be implanted as soon as there is a hemodynamic or a respiratory failure despite optimal medical management and withdrawn when possible. Table 1 summarizes the types and indications of ECMO.

### 3.5. Early Prediction of Primary Graft Dysfunction

Early pulmonary graft dysfunction (PGD) remains a major concern for transplantation teams. Recent years have seen a modification of its definition and of its hyper-early diagnosis possibilities.

#### 3.5.1. Definition

PGD results from multiple factors: the donor’s factors, preservation of the lungs, intraoperative factors (volume loading, transfusion, inspired oxygen concentration…), as well as factors related to early postoperative management [59]. Its definition was redefined in 2017 [60]. In particular, the use of ECMO for hypoxic indications with bilateral pulmonary edema on chest radiograph is graded 3 PGD while ECMO use for non-hypoxic indications without pulmonary edema on chest radiography is considered ungradable. Most studies have reported a frequency of grade 3 PGD in the range of 20–30% [60]. A grade 3 PGD at 72 h is associated with an increased risk of 90-day and 1-year mortality [61].

#### 3.5.2. Prediction

It is of prime importance to predict the risk of grade 3 PGD as early as possible to implement measures, such as ECMO. 

Numerous cohort studies have shown the importance of donor-related risk factors for PGD occurrence, notably donor age, smoking status, chronic alcohol use, cause of death, and hemodynamic instability in the peri-mortem period [62]. However, the results of these studies are sometimes contradictory as they are sensitive to the period of the study, the type of recipients and the practices of each center. 

Venkata-Subramani et al. recently proposed a conceptual framework that suggests that donors’ aging, smoking, and chronic alcohol use can independently or together promote oxidative stress and aberrant extracellular matrix remodeling, rendering the donor lung susceptible to injury and disrepair after ischemia-reperfusion triggered during organ procurement and transplantation [63]. A new way to approach this question is to look for a diagnostic test, that can be used either on the donor or during the operative period. Verleden et al. reported that the levels of IL-6 and IL-8 in the broncho-alveolar lavage are associated with an increased incidence of grade 3 PGD [64]. Similarly, complement activation fragments are detected in the broncho-alveolar lavage within 24 h after lung transplantation and are increased in patients with PGD [65]. Even more interesting, because earlier, is the analysis of pulmonary volatile organic compounds from bronchial washing and aspirates obtained immediately after surgery as recently reported by Stefanuto et al. in 35 lung transplant recipients, of whom ten will develop a grade 3 PGD [66]. Of note, Sage et al. proposed an inflammation score based on IL-6 and IL-8 levels in perfusate of ex vivo lung perfusion to identify lungs more likely to develop primary graft dysfunction at 72-h post-transplant [67], Keller et al. showed that levels of percentage donor-derived cell-free DNA on Day 1 are higher in PGD patients than non-PGD patients (*p* = 0.01) [68], and Chacon-Alberty identified a unique protein pattern in patients who did or did not develop grade 3 PGD at T48–72 h [69]. But the practicality of such measurements is far from certain, unlike the simple measure of intraoperative blood lactate level as reported in a monocenter study which brought together 449 patients, 16% of them patients having a grade 3 PGD at day 3. In this study, a value below the threshold of 2.6 mmol/L at the end of surgery has a high negative predictive value for the occurrence of a grade 3 PGD at day 3 [70]. Finally, a recent study confirmed the data of an earlier one which had put forward the predictive role of an early measurement of extravascular lung water index [71,72].

#### 3.5.3. Conclusions

Since there is no validated treatment once grade 3 PDG is present [73], the best way to prevent its occurrence is to select an optimal donor for the recipient; to optimize intraoperative strategy, to reduce inflammation (especially for hemodynamic impairment); and to identify those who require aggressive postoperative care, or early rehabilitation after surgery. New methods using a machine-learning approach are probably reliable options to identify, at a very early stage, even before leaving the operating room, patients at risk of developing grade 3 PGD three days after the surgery.

### 3.6. Pain Management

Lung transplantation candidates often suffer from preoperative chronic pain [74] and lung transplantation is a source of acute and chronic postoperative pain.

#### 3.6.1. Treatment of Early Postoperative Pain

All patients must receive an effective treatment of pain, mainly based on regional analgesia combined with a multimodal approach administered as quickly as possible orally. Such care is a prerequisite for a successful implementation of early rehabilitation after lung transplantation. However, there is moderate-quality evidence that regional anesthesia may reduce the risk of developing persistent postoperative pain after thoracotomy [75]. 

There are few randomized controlled trials comparing the numerous regional analgesic techniques in lung transplanted patients contrary to what was done after lung surgery. Thoracic epidural analgesia remains considered as the gold standard by numerous teams because of its effectiveness and association with reduced duration of mechanical ventilation and risk of respiratory complications, and shorter intensive care unit length of stay [76]. In addition, epidural anesthesia decreases sympathetic activity and stress response, has anti-ischemic effects, and improves intestinal perfusion and motility [77]. However, some teams would prefer to avoid this technique in case of probable use of mechanical circulation support, due to the risk of hematoma, or placement difficulties. In this case, chest wall blocks are a second option, less dangerous but often less effective.

McLean et al. have reported benefits of preoperative thoracic epidural placement, especially shorter postoperative ventilator time, without any neurologic complications or epidural hematomas in a population having required high anticoagulation because of the use of cardio-pulmonary bypass (CPB) in 89.5% of their patients [78]. Similarly, Fessler et al. recently reported in a monocentric cohort study of 450 transplant patients, 25% of them having had an intraoperative ECMO, that a thoracic epidural catheter was inserted in 95% of the cases, all before surgery, and that 36% were extubated in the operating room [79]. Other teams prefer the use of less invasive techniques.

As alternatives, chest wall blocks look attractive but must be selected depending on the type of surgical incision. If single lung transplantations are usually performed by a posterolateral thoracotomy, double lung transplantations may be performed by a bilateral antero-lateral thoracotomy, a clamshell incision, or a sternotomy. Thus, the cutaneous anterior branch of the intercostal nerve should be targeted in case of a sternotomy. To our knowledge, there is no publication about the transversus thoracic plane block in lung transplantation, although it has been evaluated and adopted by some anesthesiologists in cardiac surgery [80]. The cutaneous lateral branch of the intercostal nerve should be the target in case of a bilateral antero-lateral thoracotomy. There are some case reports of a serratus plane block (with or without catheter insertion) [81,82]. It seems to be a promising rescue technique, easy and safe to perform in an intensive care unit. The posterior chest wall blocks, paravertebral block and erector spinae plain block, cover all kinds of incision and are particularly appropriate in case of a Clamshell incision [83]. Hutchins et al. described a prospective cohort of 35 patients followed up to 120 h after postoperative placement of a catheter in the paravertebral space, guided by ultrasound, after lung transplantation (21 bilateral transplants and 14 unilateral transplants). The postoperative pain score was approximately around 5 over 10 from postoperative day 1 to 5. They concluded that the technique was feasible, but they did not conclude on the effectiveness for controlling pain [84]. More recently the erector spinae plane bloc has been described in a case report [85] and adopted by some teams.

Intercostal nerve block has been abandoned due to the high risk of local anesthetic toxicity, the short duration, and the difficulty of leaving a catheter in place for continuous infusion of local anesthetics. However, cryoablation of the intercostal nerve may be an attractive alternative to thoracic epidural analgesia [86]. Its prolonged analgesic effect, up to 3 to 9 months postoperatively, must be weighed against the risk of direct nerve injury (neuropathic pain and loss of intercostal muscle strength) [87]. 

Another common question is the timing of initiation of a loco-regional analgesic technique. Two situations could be opposed: patients preoperatively identified for extubation soon after surgery may benefit from an early analgesic strategy with insertion of a catheter for continuous infiltration of local anesthetics; or patients are expected to remain ventilated longer and maintained sedated. In this last case, some teams prefer to delay the regional analgesia, missing preemptive analgesia.

**Figure 1:**
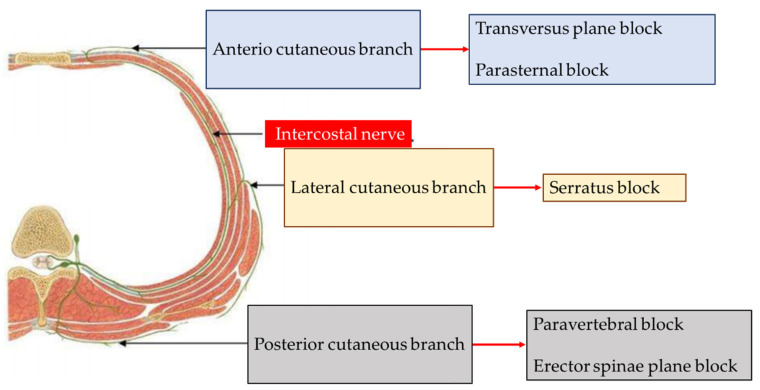
Main thoracic blocks.

Figure 1 summarizes the main thoracic blocks.

#### 3.6.2. Prevalence of Chronic Pain and Its Consequence on Quality of Life

The high prevalence of chronic pain has remained stable over the years. In a prospective observational study of 96 patients undergoing lung transplantation, Girard et al. showed that pain was present in 49% of the cases three months after surgery. The prevalence of post-thoracotomy syndrome (pain along the thoracotomy scar) was 33%. Fifty-eight percent of pain patients reported a pain score above 3 on a scale of 10, and 72% had taken analgesics on a daily or weekly basis. Furthermore, they observed that patients with a preoperative diagnosis of pulmonary emphysema were more likely to experience chronic pain [88]. Sixteen years after the paper from Girard et al., Laurent et al. observed similar results in a prospective cohort study of 72 patients undergoing bilateral lung transplantation. Six-month postoperative pain was present in 68.0% of the cases. Among patients with pain, 83.3% reported a pain score above 3 on a scale of 10, and 26.5% had neuropathic pain. However, only 9.1% took opioids. Concerning preoperative predictive factors for postoperative pain, the maximal preoperative pain score was the only independent factor identified [89]. 

In both studies, pain was associated with poor quality of life. Interestingly, Smith et al. investigated psychosocial consequences of poor quality of life as risk factors for mortality in transplant recipients. They highlighted that symptoms of depression (Beck Depression Inventory-II), and distress (General Health Questionnaire), measured 6 months after lung transplantation, were associated with increased mortality, independently of background characteristics and medical predictors. However, anxiety was not associated with increased mortality [90]. 

In addition to the usual proposed treatments for chronic pain, it can be effectively modulated through a combination of holistic pain interventions. In this setting, Michel-Cherqui et al. failed to demonstrate a significant effect of self-hypnosis on postoperative pain [91]. However, this kind of study is difficult to conduct in real life conditions. Its interest is to open the minds to other harmless techniques that may benefit the patients and to encourage other teams to join the effort and conduct a multicentric trial.

#### 3.6.3. Conclusions

If thoracic epidural analgesia tends to be replaced by chest wall blocks, less invasive techniques in usual lung surgery, this is far from being a fact after lung transplantation. Indeed, lung transplantation remains a major surgery with major incisions and major postoperative pain. This pain contributes to delayed early rehabilitation. In addition to regional analgesia and systemic painkillers, a holistic approach, including complementary techniques, should be encouraged to integrate into the patient’s management. However, these harmless techniques remain to be demonstrated in overall patient care.

## 4. Conclusions

This review shows that lung transplantation is in full evolution. However, the lack of randomized studies, particularly concerning ECMO, blood transfusion management, and pain management does not allow strong guidelines for clinicians. The low number of transplants at each center per year, and the improvements concerning outcomes can only lead to reinforce collaboration between centers to build stronger evidence-based medicine.

## Figures and Tables

**Table 1:** Types and indications of extracorporeal membrane oxygenation management (ECMO).

Types	Indications
Veno-venous ECMO	Bridge to transplantation
“	Intraoperative use
“	Postoperative use
Veno-arterial central ECMO	Intraoperative use
Veno-arterial peripheral ECMO	Bridge to transplantation
“	Intraoperative use
“	Postoperative use
Hybrid ECMO-CPB circuit	Intraoperative use

## Data Availability

Not applicable.
